# Cardiorenal Syndrome and Autonomic Overactivity

**DOI:** 10.3390/biomedicines13122947

**Published:** 2025-11-30

**Authors:** Joseph Mannozzi, Desiree Duncan, Julian D. Johnson, Andrew Kohrman, Donal S. O’Leary

**Affiliations:** 1 Department of Physiology, School of Medicine, Wayne State University, Detroit, MI 48201, USA; 2 Department of Medicine, Division of Cardiovascular Medicine, Henry Ford Health System, Henry Ford Hospital, 2799 West Grand Boulevard, Detroit, MI 48202, USA

**Keywords:** cardiorenal, heart failure, CKD, renal denervation, CSAR, baroreflex

## Abstract

Cardiorenal syndrome (CRS) is a term that describes the pathological interplay between the heart and kidney wherein either organ may be the originating stimulus that leads to acute and eventually chronic disease in the other. The mechanisms by which either initial disease progression influences the target organ are multifactorial and primarily include inflammation, alternated hemodynamics and blood volume handling, and neurohormonal alterations. The order of initiation of CRS, depending on which target organ the stimulus arises from, likely impacts the overall feed-forward mechanisms of this syndrome’s pathology; however, the end results are similar: accentuated chronic inflammation and heightened autonomic output. The latter of these symptoms of CRS is especially concerning as heightened sympathetic activity enhances the risk of various other cardiovascular events such as stroke and heart attack and ultimately limits non-pharmacological options for improving quality of life such as mild to moderate exercise. The main goal of this review is to provide an overview and outline the autonomic impacts of CRS and discuss renal denervation as a mechanism of potentially limiting or impairing the autonomic positive feedback loop initiated by disease progression and its likely subsequent amplification during exercise.

## 1. Introduction

Precise control of blood pressure and volume are the primary variables that can be modified to ensure proper cardiovascular performance, at rest or during exercise. Conditions that perturb hemodynamics most certainly initiate activation of compensatory mechanisms that are primarily driven by neurohormonal activity which ultimately affect the systemic vasculature as well as overall cardiac function. One condition that arises as a result of compensatory effects of blood pressure and blood volume control is known as cardiorenal syndrome (CRS). This syndrome describes an impaired interaction between the heart and kidneys that in concert generally leads to worsening function in both organs and has been associated with a high mortality incidence even in Type I CRS, the least chronic condition [1]. As the name suggests, CRS arises from feedback-related stress that can occur initially from either the heart or the kidney through significant alteration in the hemodynamic environment that in turn impairs the heart or kidney depending on where the originating stimulus arises; this has led to a classification system of the disease as a whole [2]. For instance, acute cardiac or renal injury-mediated CRSs are classified as Type I (acute cardiac initial injury) or Type III (acute renal initial injury) whereas chronically mediated induction of CRS falls under Type II (chronic cardiac) and Type IV (chronic renal) depending on the organ from which the initial insult arises. A Type V exists but this is typically not a result of one organ acting on the other, but more of a systemic simultaneous impairment to both organs, such as amyloidosis, sepsis, cirrhosis, etc., that then is perpetuated by poor function in both organs on each other [2,3,4]. Of the five classifications, Type II CRS has the highest prevalence among the CRS etiologies [2,5,6] likely due to the chronic nature of hypoperfusion induced by chronic heart failure, but also through the long-term pharmacological treatments utilized to reduce cardiac strain. This observation is interesting as the most recent records from the CDC and AHA [7,8] show that chronic kidney disease prevalence is much higher (14%) relative to heart failure prevalence (2–4%) in the United States. Thus, given the disparity in the diagnosis of CRS types, this insinuates that the renal system is more sensitive to a cardiac insult than the cardiac system is to renal afferent feedback effects. Regardless of the origination of the insult, either scenario leads to the potential development of positive feedback loops: the response from the originally injured organ damages the second organ. The assault on the second organ feeds back onto the first organ, which amplifies output from this first organ. This further damages the second organ leading to an ever-spiraling vicious positive feedback loop scenario. The ability of these feedback loops controlling autonomic activity to remain limited in disease is impaired [9,10,11]. Thus, the goal of this review article will be to describe the feed-forward mechanisms of CRS disease development and to discuss the potential aspects of feed-forward autonomic alterations that likely assist in potentiating disease progression at rest and during exercise.

## 2. Cardiac and Renal Neurohormonal Activation

All organs exhibit some form of paracrine signaling and autonomic innervation, more commonly referred to in combination as neurohormonal signaling. Neurohormonal signaling is a diverse communication mechanism by which various organs can provide information in an integrative manner to maintain homeostasis and this is especially pertinent with regard to the cardiovascular system [12,13]. Regarding the heart itself, various feedback mechanisms sensitive to perfusion, cardiac damage, and cardiac filling pressures exist to directly modify the output of the heart itself. These response mechanisms are primarily driven by local afferent responses like the cardiac sympathetic afferent reflex (CSAR) (an amplifying stimulus in response to poor perfusion and metabolic waste accumulation) [9,10,14,15], the Bezold Jarisch reflex (a primarily parasympathetic response to ventricular ischemia that contributes to low heart rate and syncope) [16,17], and the Bainbridge reflex (a response to altered atrial stress that can modulate heart rate and therefore cardiac output) [18]. Although many other cardiovascular-related reflexes exist and impact cardiac function like the arterial baroreflex and various chemo-sensitive reflexes, these are three of the primary cardiac tissue-initiated cardiovascular reflexes.

In addition to these reflex responses that have a direct effect on altering hemodynamics, cardiac tissue also in response to stress can elicit release of atrial natriuretic peptide, which in turn leads to vasodilation and volume excretion to help manage blood pressure [19]. Under normal conditions, these intrinsic cardiac reflexes are deployed to ensure proper maintenance of cardiac function and to mitigate changes in function as a result of stress and pathology development. However, only one of these three main cardiac reflexes is known to be tonically active in pathology, propagating elevated autonomic outflow as a result of continual under-perfusion and metabolic accumulation within cardiac tissue, and that is the CSAR [9,15,20,21,22,23,24,25]. The primary effect of tonic CSAR activation is observed as elevated total vascular resistance, increased heart rate, and poor ventricular function, which all likely contribute to adverse cardiac remodeling observed in models of heart failure within intact CSARs [9,10,14,15,26,27,28,29,30] as well as alterations in baroreflex function [20,27,30]. Although the chronic effects of altered perfusion mechanics likely play a driving role in initiating pathophysiological alterations and other end-organ afferent feedback responses, the most interesting effect of CSAR activation in regard to autonomic function in cardiorenal syndrome occurs through its interaction with the arterial baroreflex. Previous studies have shown that cardiac afferent activation impairs baroreflex function [20,22,27,30]. One study by Gao et al. [20] showed that impaired baroreflex function from cardiac afferent activation directly impaired the gain or strength of baroreflex control of renal nerves [20] that could thereby be a mechanism for a vicious cycle feedback loop potentiating the development of cardiorenal syndrome by autonomic mechanisms. Indeed, studies have shown that chronic heart failure and CSAR activation increase renal efferent activity and overall renal sympathetic nerve activity [26,31,32,33,34,35]. These findings illustrate that the observation of tonic CSAR activation in heart failure [9] can influence sympathetic outflow directly to the kidney, modulating renal function via renal vasoconstriction and activating neurohormonal cascades such as renin–angiotensin system activation [36,37].

Similarly to the cardiac afferent reflex, renal afferent activation in the absence of overt heart failure can have wide-ranging effects on various target organs via mediating general increases in total sympathetic outflow [38]. That said, in the context of the neurohormonal interaction between the heart and the kidney, renal afferent and efferent activity alters cardiac function in two distinct mechanisms: 1. increased sympathetic activity to the heart and systemic vasculature [32,33,38,39,40,41]; and 2. activation of the renin–angiotensin system centrally and peripherally leading to global increases in blood pressure and altered autonomic signaling [42,43,44,45]. Together, these effects work in concert increasing cardiac stress either through direct enhanced autonomic outflow, or through driving significant increases in afterload, both of which can induce negative shifts in cardiac function when prolonged. Thus, pre-existing renal afferent and efferent activation, via acute kidney injury, chronic kidney disease, or other comorbid kidney stressor such as prediabetes, obesity, etc., may be a mechanism by which neurohormonal activation from the kidney can impede cardiovascular function, or at a minimum amplify existing sympathetic outflow and lead to altered cardiac function. However, whether or not increased renal nerve activity in heart failure is a result of renal injury or an effect of renal efferent alteration in renal function is not well understood, though the end effect is essentially the same in that sympathetic activity is amplified and has negative effects on both cardiac and renal function.

## 3. Cardiorenal Positive Feedback Loop Scenarios

### 3.1. Type III and IV Renal-Initiated CRS

Although disparities exist regarding the diagnosis of renal-mediated CRS relative to cardiac-initiated disease, these differences do not necessarily reduce the significant involvement of the kidney in CRS progression. The contribution of the renal-initiated subtypes to the overall incidence of CRS is reduced due to the low prevalence of acute kidney injury (AKI) [46]. AKI in isolation is generally caused by correctable conditions such as hypovolemia, toxins, and/or sepsis, [47,48,49,50,51] and thus it is likely that the long-term systemic burden required to alter cardiovascular function is not maintained long enough to reach CRS. Furthermore, the association of AKI with CRS is likely a result of the comorbid cardiovascular deficit as a recent study has shown that AKI increases by 50% in ICU patients, and of those individuals with AKI, 41% of those patients were admitted for cardiovascular complications prior to developing AKI [51].

Chronic kidney disease (CKD) is the initiator of Type IV CRS and has a higher prevalence than Type III AKI-induced CRS likely due to the longer duration of inflammatory, neurohormonal, and hemodynamic abnormalities elicited by the kidney in this chronic progression. In either condition, whether it be AKI or CKD, the resulting effects on cardiovascular function are generally the same wherein systemic renin angiotensin system activation combined with increased renal afferent activity and altered electrolytic balance lead to robust alterations in the heart’s ability to provide adequate systemic perfusion. These mechanisms of renal signaling generally lead to cardiovascular impairment through significant increases in afterload but can also influence fibrosis patterns and overall blood pressure and circulatory volume control [38,40,44,52,53,54]. Consequently, some of these aforementioned effects that are more short-term potentially contribute to the altered myocardial efficiency observed in CKD patients. Individuals with CKD in the absence of left ventricular geometry changes have poor myocardial work efficiency compared to control individuals [55,56]. Furthermore, as the symptoms of CKD progress further or are combined with alterations in ventricular morphology, myocardial work is not only inefficient, but work capacity also becomes impaired [55,56,57]. Myocardial work is an assessment of myocardial efficiency, not too dissimilar to stroke work and cardiac power which pertain to ventricular efficiency and provide information related to cardiac output and myocardial oxygen supply–demand relationships [55,58,59,60,61,62]. Therefore, it is likely that even impaired myocardial efficiency is sufficient to further perturb hemodynamics either through direct effects on the heart itself or by way of impairing ventricular energy transfer to the systemic circulation [61,63,64,65]. Thus, as ventricular function adapts and or becomes impaired due to the initial renal-mediated insult, the feed-forward nature of CRS is initiated, as well as the potential potentiation of exaggerated autonomic activity. With these altered hemodynamics, the combined neurohormonal effects of the renin–aldosterone–angiotensin system (RAAS) and increases in sympathetic activity are likely significant potentiators of disease.

At present, there are multiple mechanisms of interrupting and/or altering the RAAS, and the use of these pharmaceutical interventions is beginning to gain ground clinically for the treatment of CRS in addition to treatments aimed at altering blood volume such as loop diuretics [5,66,67,68]. Conversely, few therapeutic interventions currently exist that would combat renal or cardiac afferent feedback that could perpetuate sympathetic nervous system dysfunction. Renal afferents are generally sensitive to disturbances in the kidney involved in mechano-transduction and metabolite sensitivity, although little is known about the exact mechanisms of stimulation that alter afferent signaling [44,69]. That said, once activated, these afferents have been shown to elicit robust sympathetically mediated alterations in blood pressure, heart rate, and vascular function [32,38,40,69,70]. Interestingly, one mechanism by which this sympathetic overactivity may be potentiated in CKD is baroreflex dysfunction, which has been observed to be present in both juvenile and adult CKD animals [71] as well as in humans [72]. Whether or not this baroreflex dysfunction is potentiated via renal afferent or hormonal mechanisms has yet to be determined. To date, the impact of renal afferents on modifying arterial baroreflex function has not been well evaluated with most studies showing a generally reduced sympathetic outflow and improved peripheral baroreflex activity as a result of renal denervation (RDN), but cardiac baroreflex activity and efferent renal nerve activity may not follow suit and may be impaired or altered by denervation [32,40,42,73,74].

In addition to and potentially in concert with altered arterial baroreflex function, cardiac sympathetic afferent reflex (CSAR) activation has been observed in CRS, and its attenuation improves renal function [26]. Cardiac sympathetic afferents are located within the myocardium and respond to a plethora of stimuli including those associated with poor myocardial oxygen delivery which are often observed in diseases leading to enhanced sympathetic outflow [9,15,21,23,24,27,75,76]. Although likely the potentiating stimulation in CRS Type II, CSAR is likely also significantly active in CRS Type IV. Previous observations have shown significant reductions in myocardial work in CKD [55,56,57]. These reductions in concert with the impacts of RAAS activation on cardiac sympathetic afferents [22] may be sufficient to activate the CSAR and therefore further exaggerate the existing sympathetic overactivity in CKD, thereby potentiating the progression into CRS. Another interesting observation is that the CSAR has been shown to inhibit baroreflex activity in heart failure [22,29,30]. Impaired baroreflex function as a result of CSAR activation thereby provides an additional mechanism of feed-forward sympathetic overactivity in CRS.

### 3.2. Type I and II Cardiac-Initiated CRS and Off-Target Organ Effects

Unlike insults from the kidney, cardiac-mediated CRS is far more prevalent in the general population [2,3], even though the primarily diagnosed version Type II originates from heart failure which has a much smaller incidence rate than CKD alone [7,8]. However, a 2014 meta-analysis revealed that 49% of heart failure patients had some form of CKD and that 25–35% had AKI [77]. The effect of variation in insult may insinuate that the heart itself is far more resistant to insult relative to the kidney, or that hypoperfusion as a result of cardiac injury or impairment is a very potent stimulus for activation of renal neurohormonal activation. Regardless of which primary insult is more significant, the cascade remains relatively the same, and in this case, with either form of cardiac-mediated CRS, the primary stimulus for initiation of the disease is poor cardiac performance leading to under-perfusion of the kidney [3,5,6,78]. Under-perfusion of the kidney is combated typically via three mechanisms: renal autoregulation, RAAS activation, and increased renal afferent activity that alters autonomic function [79]. Once activated, these mechanisms work to correct the impaired renal perfusion, but at a cost, by way of further exacerbating existing cardiovascular dysfunction. Activation of the RAAS leads to a significant increase in cardiac afterload via vasoconstriction, increased circulatory volume, and increased CSAR activity [22]. Together, these in turn likely lead to an increase in cardiac work and thereby elicit a greater myocardial oxygen demand. If myocardial oxygen demand is not met with myocardial oxygen supply, then CSAR activation will become even more exacerbated, leading to even greater sympathetic activation that in turn will impair renal blood flow but also further offset the myocardial oxygen supply–demand relationship and thus the cycle will continue. As this cycle perpetuates forward, the impact on the arterial baroreflex likely becomes magnified overtime leading to even greater and greater loss of baroreflex-mediated restraint of RAAS and CSAR, thereby potentially setting the stage for a sympathetically mediated major cardiac event during strenuous activity or exercise.

Recent studies have shown that cardiac sympathetic afferent activation in heart failure is chronic and may lead to the exaggeration of sympathetic responses during exercise and contributes to arterial baroreflex impairment [9,15,27,29,80,81]. In conditions such as CRS I and II, the CSAR is also likely active and has been for some time (Type I CSAR due to existing cardiovascular dysfunction in the absence of heart failure). However, due to the initial insult being from the heart, the use of ablative techniques to attenuate CRS progression may be difficult unless proactive at the onset of diagnosis. CSAR ablation is not yet an accepted clinical therapy for attenuation of exaggerated cardiovascular autonomic activity. However, RDN is a technique that has been used in recent clinical trials to combat resistant hypertension and therefore provides a potential avenue for treatment of this self-perpetuating autonomic feedback loop [32,38,42,44,82,83].

The primary theory behind the use of RDN in the most recent clinical trials of hypertension was that by impairing renal afferent and efferent activity, the kidney would no longer be able to potentiate neurohormonal activity that maintains hypertension. The loss of renal input systemically would in turn elicit a systemic reduction in sympathetic activity both through reduced renal afferent input, as well as through reductions in global Ang II receptor activation [83]. Furthermore, over time, the reductions in systemic neurohormonal signaling may lead to reductions in existing or future cardiac and vascular fibrosis [12,27,84]. In the absence of exaggerated autonomic activity, there is also the potential to protect additional organ systems affected by any type of CRS. One potentially affected system especially in late-stage disease is the respiratory system. In CKD and heart failure, the respiratory system is subject to improper volume handling perfusion via systemic venous congestion secondary to either the initial heart failure or CKD in CRS [85,86,87]. This systemic congestion leads to an increase in interstitial fluids especially in respiratory tissue, which in turn impairs the movement of blood through the pulmonary system and alters oxygen and carbon dioxide perfusion gradients and, with that, impairs the ability of the lungs to assist with acid-base handling [88,89]. This combined effect presents clinically as dyspnea and shortness of breath, which in turn can lead to further sympathetic activation via altered chemoreflex activity and enhanced respiratory muscle fatigue [90,91,92]. All of these respiratory issues could be exacerbated during even mild to moderate exercise [93]. Although not studied in depth, exploring the autonomic and neurohormonal impacts of CRS on various other organ systems beyond the heart and the kidney is warranted as in cases such as that with the respiratory system, the contribution of autonomic function and altered homeostatic functions of a given organ may magnify an existing poor prognosis in CRS.

## 4. Conclusions and Future Directions

**Figure 1:**
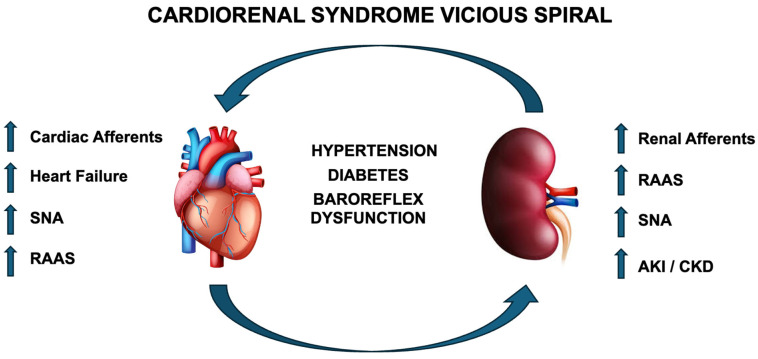
Illustration of positive feedback loop of CRS originating from either the heart or kidney. RAAS—renin–angiotensin–aldosterone system; SNA—sympathetic nerve activity; AKI—acute kidney injury; CKD—chronic kidney disease.

CRS is an insidious multifactorial pathology likely potentiated by positive feedback amplification, as the heart and kidney progressively affect each other with negatively spiraling dysfunction, leading to often extreme cardiac and renal impairment. This vicious spiral is summarized in Figure 1. In addition to the robust neurohormonal and hemodynamic effects of CRS, the aberrant activation of autonomic reflexes as a whole potentiates dysfunction, thereby making treatment options to improve CRS limited. Although there are multiple potential avenues for improving outcomes in CRS, based on the current literature, one promising avenue would be to treat the kidney, which would have significant benefits for all types of CRS. For instance, in cardiac-initiated CRS, the kidney is affected secondarily; thus, at the time of disease induction, if kidney function could be preserved, this could prevent a positive feedback loop. In renal-mediated CRS, the kidney itself is the initiator, and thus, if renal function can be preserved at the onset of overt cardiac pathology, cardiac damage may be limited, thereby preventing initiation of a vicious cycle (Figure 1), which could limit further hemodynamic instability by preserving cardiac function. Multiple options exist that impact blood volume control such as diuretics, glycosuria-inducing agents, RAAS inhibitors, and many others. Each of these options require habitual patient compliance and access to healthcare that is not always available in all socioeconomic situations. Thus, a more appealing option would be the potential administration of a one-time procedure such as RDN that could alleviate disease potentiation and reduce disease symptoms.

RDN therapy has been shown to reduce blood pressure in hypertensive patients resistant to current pharmacologic therapies [94,95]. One of the primary effects of RDN is a reduction in overactive sympathetic tone, which has been shown to contribute to a myriad of disorders such as HTN, CKD, and HF, all of which can contribute to the development of CRS [10,12,21,42,44,69,70,96,97,98,99]. In each of these conditions, aberrant sympathetic activity is a factor, and the ability to limit that activity is a crucial component in order to minimize disease progression. Given that RDN is a certified intervention by the FDA and is known to reduce blood pressure primarily through disrupting renal-mediated sympathetic activation [38,69,95], it presents an existing mechanism to fight aberrant autonomic function in all the aforementioned conditions but also especially in CRS.

Although not yet evaluated in CRS directly, various clinical trials are underway evaluating the efficacy and safety of RDN in heart failure patients as a mechanism to reduce autonomic burden and cardiac disease progression. The heart failure-specific trials come on the heels of hypertension trials that offered mixed results. The SYMPLICITY 3 trial results at 6 months showed minimal therapeutic benefit and potentially biased benefits at 3-year follow-up [100,101]. However, a meta-analysis evaluating the effects of RDN over the duration of all of the SYMPLICITY trials was able to illustrate that RDN had positive effects on blood pressure control [102]. Similarly to studies in animals on hypertension, studies in various animal models of heart failure evaluating the effects of RDN illustrate primarily positive results such as improvements in ejection fraction, strain, cardiac remodeling/fibrosis, volume handling, neurohormonal activation, and vascular function [103,104,105,106,107]. However, the translation of these studies to humans has had mixed results. Furthermore, while RDN is an approved therapeutic intervention for the treatment of hypertension, it is not approved yet for the treatment of either heart failure with preserved or reduced ejection fraction.

The first initial study observing the effect of RDN in humans with heart failure with reduced ejection fraction (HFrEF) was conducted in 2013 and illustrated that RDN in HFrEF patients did not demonstrate statistically significant benefits in regard to blood pressure, or standard echocardiographic metrics of cardiac function [108]. However, the primary goal of this study was an evaluation of safety, and thus longer-term studies were warranted to evaluate cardiac improvements beyond the reported improvements in exercise tolerance [108]. From here, a myriad of studies were conducted both as random and non-random controlled trials. Subsequently, two meta-analyses performed in 2020 and 2023 illustrate significant clinical benefits outlined by significant improvements in ejection fraction, six-minute walk distance, nt-proBNP, heart rate, as well as atrial and ventricular characteristics [109,110]. In contrast to studies in HFrEF, observations in heart failure with preserved ejection fraction (HFpEF) utilizing RDN have yielded varying results across both animal and human studies. Some of the variation in animal model results may be a result of species-specific effects and the mechanisms of HFpEF induction [111,112,113,114,115], and the timing in which RDN is applied. With regard to timing, a recent study in a preclinical model of mini pigs observed that application of RDN has no significant effect on long-term blood pressure reductions in HFpEF [116], and an additional study in rodents observed that early treatment but not late treatment with RDN in HFpEF was more efficacious [111]. The observed lack of continuity extends to studies in humans likely as a result of the wide variation in associated comorbidities and mechanisms of development that can lead to inappropriately diagnosed HFpEF [117,118,119]. At present, even with the difficulties associated with the diagnosis of HFpEF, one random controlled clinical trial and four observational studies utilizing patient records from previous trials and cohorts have shown promising results that RDN improves diastolic function, reduces adverse cardiac biomarkers, and most importantly appears to improve quality of life [120,121,122,123]. Overall, the impact of RDN as a therapeutic intervention in either type of heart failure appears to, at a minimum, improve exercise capacity and metrics of life quality. However, significant limitations were viewed across studies primarily in regard to standardization and timing. Future studies involving HFrEF, should seek to investigate the benefits of RDN earlier in systolic dysfunction progression while also addressing the impact of variation among classification of heart failure and time to follow-up assessment. Alternatively, future studies on HFpEF should seek to normalize the diagnostic criteria of HFpEF included in studies as well as time since diagnosis. The latter of these two issues may prove to be the most important as models of HFpEF in animals have shown that earlier treatment windows may be more beneficial than in Frank disease [111,124]. With regard to CRS, RDN as a therapeutic tool has yet to be fully investigated in human patients, and the data in animals at present is more linked to the use of RDN for treatment of existing pathologies such as heart failure and chronic kidney disease, two drivers of Type II and Type IV CRS. We suggest that study designs should aim to ensure that the types of heart failure and mechanisms of staging it, such as the Kansas City Cardiomyopathy Questionnaire and the NYHA class diagnosis, are as even as possible across patient populations. From a renal perspective, studies on RDN and CRS should implement recruitment of subjects with similar renal function patterns and potentially look to treat subjects in the earlier stages of cardiovascular impairment that is comorbid with underlying renal disease. Finally, it may be prudent to evaluate underlying autonomic dysfunction, as the primary goal of renal denervation is to eliminate autonomic feedback and amplification. Subjects lacking significant autonomic amplification may not be appropriate for inclusion as the removal of renal afferent activity may provide no additional benefits to these patients from a study metrics prospective, even if they may receive the benefit of protection from development of renal-mediated autonomic amplification.

The ability of RDN to alleviate symptoms of CRS is yet to be investigated. This may be potentially important especially in patient populations that have traditionally been medically underserved and disproportionately burdened by cardiovascular comorbidities, like CRS. The link between cardiovascular disease and the social determinants of health has been well-studied [125]. To better understand this relationship and the implications of RDN in potentially mitigating healthcare disparities, the African American population serves as a critical framework. African Americans (AA) have generally been disproportionately burdened by CRS, as well as with the pre-existing conditions associated with CRS development (HTN, CKD, and HF) [126,127,128,129]. More specifically, AAs have higher rates of heart failure, chronic kidney disease, diabetes, HTN, as well as higher rates of associated comorbidities when compared to their white counterparts [130] and a higher relative rate of heart failure-related mortality compared to their peers. In addition, Black patients have one of the highest rates of HF-related death compared to other races [131]. Therefore, when discussing the primary goal of advancing health equity, it is important to consider the underlying dynamic environment and patient outcomes and pair adaptive efficacious solutions that potentially are a better fit then traditional therapeutic interventions. For instance, the use of RDN as a potential treatment for CRS offers a solution to multiple barriers that exist in providing care to patients with complex medical history and socioeconomic constraints. Some of these barriers to care are (1) medication adherence and access; (2) long-term cost; (3) potential provider bias; and (4) burden of polypharmacy; all of these can be relatively corrected by the one-time nature of an RDN procedure. Thus, RDN has the potential to limit systemic inequities, alleviate disease burden, and improve outcomes for patient populations with heart failure that have traditionally been overlooked in the past.

In conclusion, the use of RDN as a therapeutic intervention for CRS presents an interesting avenue to limit at least one aspect (neurohormonal/autonomic dysfunction) of the various positive feedback loops likely active in this disease. Furthermore, RDN as a treatment may be able to improve disease burden in outcomes in patient populations disproportionally affected by components that lead to CRS. Future studies in animals and humans should seek to normalize the types of disease burden and identify existing autonomic dysfunction prior to treatment. In some of the clinical trials as well as animal studies, one potential confounding mechanism may be a lack of sufficient autonomic dysfunction to induce identifiable therapeutic benefit by removal of renal nerve-mediated autonomic amplification with RDN. Furthermore, in animals, it would also be pertinent to further explore the use of RDN as a pretreatment for CRS as this may be a mechanism of attenuating CRS development that could be applied in humans with cardiac or renal impairment in the absence of overt comorbidities. Regarding CSAR and CRS, many questions remain unanswered as to the level of activation in CRS and CRS development, if attacking the CSAR would be a viable mechanism of reducing autonomic tone and thus limiting renal injury, and finally if CSAR modulation is even a viable strategy in humans given the complex nature of the innervation of the heart. Overall, CRS remains a difficult condition to treat as it is multifactorial and originates from various pathologies that appear autonomically driven. Entertaining and employing innovative therapies such as RDN for CRS treatment engender a mechanism of treating autonomic amplification, but also potentially a pathway to fighting socio-economic barriers to healthcare for the patient populations most likely affected.

## Data Availability

No new data were created or analyzed in this study. Data sharing is not applicable to this article.
